# Supplementary material for: Oral Cancer-Derived miR-762 Suppresses T-Cell Infiltration and Activation by Horizontal Inhibition of CXCR3 Expression
Source: Int J Mol Sci. 2025 Jan 26;26(3):1077. doi: 10.3390/ijms26031077 (PMC11817288; doi:10.3390/ijms26031077)
Supplement: Supplementary file 1 [file ijms-26-01077-s001.zip › ijms-3337590-supplementary.pdf]

Table S1 **Cell line background**

Cell Name	Cell bank	Catalogue Number	RRID
Jurkat	BCRC	60424	CVCL_0065
293T	ATCC	CRL-3216	CVCL_0063
SCC-4	ATCC	CRL-1624	CVCL_1684
SCC-9	ATCC	CRL-1629	CVCL_1685
SCC-15	ATCC	CRL-1623	CVCL_1681
SCC-25	ATCC	CRL-1628	CVCL_1681
Cal-27	ATCC	CRL-2095	CVCL_1107
Cal-33	DSMZ	ACC 447	CVCL_1108
OEC-M1	NDMC		CVCL_6782
HSC-2	JCRB	JCRB0622	CVCL_1287
HSC-3	JCRB	JCRB0623	CVCL_1288
HSC-3-M3	JCRB	JCRB1354	CVCL_8323
HSC-4	JCRB	JCRB0624	CVCL_1289
Ca9-22	JCRB	JCRB0625	CVCL_1102
OSC-19	JCRB	JCRB0198	CVCL_3086
OSC-20	JCRB	JCRB0197	CVCL_3087
SAS	JCRB	JCRB0620	CVCL_1675

Primers

Gene Name	Forward primer	Reverse primer
CD69	ATTGTCCAGGCCAATACACATT	CCTCTCTACCTGCGTATCGTTTT
IL2RA	CGCAGAATAAAAAGCGGGTCA	ACTTGTTTCGTTGTGTTCCGA
CD71	GGCTACTTGGGCTATTGTAAAGG	CAGTTTCTCCGACAACTTTCTCT
CXCR3A	ACCCAGCAGCCAGAGCACC	TCATAGGAAGAGCTGAAGTTCTCCA
CXCR3B	TGCCAGGCCTTTACACAGC	TCGGCGTCATTTAGCACTTG
CXCR3 alt	CCAATACAACTTCCCACAGGGGT	GTCTCAGACCAGGATGAATCCCG
GAPDH	GAAGGTGAAGGTCGGAGT	GAAGATGGTGATGGGATTTC
miR-762-F	TTATGGGGCTGGGGCCGG	
U6-F	CGCAAGGATGACACGCAAATTC	
CXCR3-	CGGACCGGGCCGGAATCCGGGCTCCCCTTTC	CGGTCCGTCCTGACGATCTTGTTTATT

UTR-cloning		
Mutation miR-762 MRE on CXCR3-UTR	AGGGTGCTGCACAATGAAGCGACAACGCAGGCCTCCAGCTC	CTGGAGGCCTGCGTTGTCGCTTCATTGTGCAGCACCCTCTA

Antibody list

Gene name	Brand	Catalogue Number
Total-AKT	Cell Signaling	4691
phospho-AKT (pS473)	Cell Signaling	4060
CXCR3	ABclonal	A11294
GAPDH	GeneTex	GTX100118
CD25-PE	BD Biosciences	341009
CD69-PE	BD Biosciences	341652

Chemical reagents

**Name**	**Brand**	**Catalogue number**
TransIT-X2	Mirus	MIR 6000
Blasticidin	Invivogen	ant-bl-05
Fibronectin	Sigma	F1141
GeneArt™ site-directed mutagenesis system	Invitrogen	A13282
HE swift cloning kit	Biotools Co.	TB-VTT-BB05
Hoechst 33342	Invitrogen	H3570
Human IL12 ELISA kit	R&D	D1200
Immobilon western chemiluminescent HRP substrate	Millipore	WBKLS0500
Lipofectamine 2000	Thermo	11668030
LR Clonase II plus enzyme	Invitrogen	12538-200
ChamQ Universal MasterMix	Vazyme	Q711
ONE-Glo™ Luciferase assay system	Promega	E6110

Pierce™ BCA Protein Assay Kit	Thermo	23225
Puromycin	Invivogen	ant-pr-1
NxtScript Reverse Transcriptase	Roche	07051166103
TRIzol™ Reagent	Invitrogen	15596026
miScript II RT Kit	QIAGEN	218160
anti-CD3 antibody (Clone:OKT3)	BioLegend	317353
anti-CD28 antibody (Clone: CD28.2)	BioLegend	302977100
IL-12	R&D	219-IL
CCK8 Kit	dojindo	CK04
miR-762	Genomics	MI0003892
miR-NC	Genomics	
jetPEI	PolyPlus	101000053

Vectors

**Name**	**Brand**	**Catalogue number**
7TFP-CDH1 reporter	Addgene	91704
pGreenFire2.0-NFkB	SBI	TR412PA-P
pDONR221-CXCR3	DNASU	HsCD00438455
pLenti6.3-DEST	Invitrogen	V53306
pGreenFire-CMV-CpoI	SBI	TR011PA-1

Supplementary Figure S1

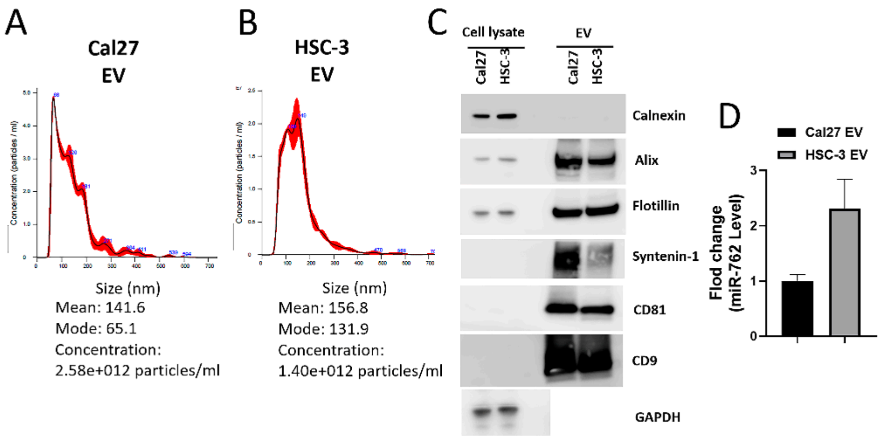

miR-762 is OSCC exosomal microRNA. A & B. Cal-27 (A) and HSC-3 (B) exosome particle size analysis from NTA. C. Expression of exosome markers from purified OSCC exosomes. D. exosomal miR-762 expression level from OSCC exosome.
